# *SigFlux*: A novel network feature to evaluate the importance of proteins in signal transduction networks

**DOI:** 10.1186/1471-2105-7-515

**Published:** 2006-11-27

**Authors:** Wei Liu, Dong Li, Jiyang Zhang, Yunping Zhu, Fuchu He

**Affiliations:** 1 College of Mechanical & Electronic Engineering and Automatization, National University of Defense Technology, Changsha, China; 2 Beijing Institute of Radiation Medicine, No.27 Taiping Road, Beijing, China; 3 Beijing Proteome Research Center, No 33 Life Science Road Changping District, Beijing, China

## Abstract

**Background:**

Measuring each protein's importance in signaling networks helps to identify the crucial proteins in a cellular process, find the fragile portion of the biology system and further assist for disease therapy. However, there are relatively few methods to evaluate the importance of proteins in signaling networks.

**Results:**

We developed a novel network feature to evaluate the importance of proteins in signal transduction networks, that we call *SigFlux*, based on the concept of minimal path sets (MPSs). An MPS is a minimal set of nodes that can perform the signal propagation from ligands to target genes or feedback loops. We define *SigFlux *as the number of MPSs in which each protein is involved. We applied this network feature to the large signal transduction network in the hippocampal CA1 neuron of mice. Significant correlations were simultaneously observed between *SigFlux *and both the essentiality and evolutionary rate of genes. Compared with another commonly used network feature, connectivity, *SigFlux *has similar or better ability as connectivity to reflect a protein's essentiality. Further classification according to protein function demonstrates that high *SigFlux*, low connectivity proteins are abundant in receptors and transcriptional factors, indicating that *SigFlux *candescribe the importance of proteins within the context of the entire network.

**Conclusion:**

*SigFlux *is a useful network feature in signal transduction networks that allows the prediction of the essentiality and conservation of proteins. With this novel network feature, proteins that participate in more pathways or feedback loops within a signaling network are proved far more likely to be essential and conserved during evolution than their counterparts.

## Background

Structural analysis of signal transduction networks can provide insight into the function and evolution of the cellular networks. A proper network feature to evaluate the importance of each protein in signaling networks helps to identify the crucial proteins in a cellular process and further provides us with a better understanding of complex diseases and a guiding principle for therapy design. Relatively few methods [1-4] have been proposed so far to analyze the structure of signaling networks. Particularly, a software tool *CellNetAnalyzer *was developed in [2] to compute feedback cycles and all the signaling paths between any pair of nodes, but these cannot be used to evaluate the importance of each protein in signaling networks.

In another front, the structural analysis of metabolic networks has been well studied, although these studies have been scarcely applied to signaling networks. The method to evaluate the importance of enzymes in metabolic networks is based on the concept of elementary flux modes (EMs) [5,6]. EMs are minimal sets of enzymes that can operate at steady state. The number of elementary modes in which an enzyme is involved assesses the importance of the enzyme [7]. Elementary mode analysis appears to be well-suited to characterize network properties because each elementary mode is non-redundant. However, the algorithm for EM calculation cannot be used to signal transduction networks directly because the computation of elementary modes in a given network requires the stoichiometric matrix and the reversibilities of the reactions. While in large signaling networks, the construction of precise quantitative models is practically infeasible due to the huge amount of required but generally unavailable kinetic parameters and concentration values [8,9].

Connectivity [10] and the clustering coefficient [11] are two well-known topological characteristics describing the importance of a protein in protein interaction networks. Connectivity of a node is the number of its interacting partners, and the clustering coefficient defines the cliquishness of each node. Proteins with high connectivity and clustering coefficient tend to be essential in protein interaction networks [10,12]. However, it is unknown whether these two characteristics are also suited to measure the importance of proteins in signaling networks.

In this paper, we introduce a concept of minimal path sets (MPSs) to measure the importance of proteins in signaling networks. An MPS in signal transduction networks can be considered as a minimal set of proteins functioning together to perform signal propagation. MPSs are inherent and uniquely determined structural features of signaling networks similar to EMs known from metabolic networks. The conceptual properties of MPSs offer a number of potential applications both for obtaining a deep understanding of structural properties of cellular networks as well as for finding targets that efficiently activate or inhibit cellular functions. Based on MPSs, we further propose a network feature, which we call *SigFlux*, to assess the importance of each protein. We examined the usefulness of *SigFlux *for assessing the importance of proteins in the signaling network of the mouse hippocampal CA1 neuron [13] using mutant phenotype and the evolutionary rate of mouse genes. We compared the performance of *SigFlux *with two other network features, connectivity and the clustering coefficient.

## Results

### A novel network feature reflecting a protein's importance

Similar to the definition of elementary flux modes in metabolic pathway analysis, an MPS in signal transduction networks refers to a minimal set of proteins that can propagate the signal from input to output, while regarding the extracellular ligand as input and the finally regulated gene in the nucleus as output. In addition, feedback loops, which were suggested in [14] to have a specific biological function, widely exist in signaling networks and can be regarded as another type of MPSs.

One important application of EMs in metabolic networks is to evaluate the importance of one or a set of enzymes. Similarly, MPSs facilitate the assessment of the importance of each protein in a signaling network. In this paper, we develop a C++ program for computing MPSs by generating all the paths between input and output using the breadth-first search method [15]. Feedback loops in the networks are identified using MFinder [16,17] and also counted as MPSs. Because feed-forward loops have already been counted in the computation of paths between the input layer and the output layer, there is no need to count them separately.

We propose the following *SigFlux *measurement to assess the importance of each protein in signaling networks. For each protein, we define *SigFlux *to be proportional to the number of all MPSs in which the protein is involved. More precisely, *SigFlux *of protein *i *is defined as

SigFlux=mpi+mfi∑i=1n(mpi+mfi),     (1)
 MathType@MTEF@5@5@+=feaafiart1ev1aaatCvAUfKttLearuWrP9MDH5MBPbIqV92AaeXatLxBI9gBaebbnrfifHhDYfgasaacH8akY=wiFfYdH8Gipec8Eeeu0xXdbba9frFj0=OqFfea0dXdd9vqai=hGuQ8kuc9pgc9s8qqaq=dirpe0xb9q8qiLsFr0=vr0=vr0dc8meaabaqaciaacaGaaeqabaqabeGadaaakeaacqqGtbWucqWGPbqAcqWGNbWzcqWGgbGrcqWGSbaBcqWG1bqDcqWG4baEcqGH9aqpdaWcaaqaaiabd2gaTnaaBaaaleaacqWGWbaCcqWGPbqAaeqaaOGaey4kaSIaemyBa02aaSbaaSqaaiabdAgaMjabdMgaPbqabaaakeaadaaeWbqaaiabcIcaOiabd2gaTnaaBaaaleaacqWGWbaCcqWGPbqAaeqaaOGaey4kaSIaemyBa02aaSbaaSqaaiabdAgaMjabdMgaPbqabaGccqGGPaqkaSqaaiabdMgaPjabg2da9iabigdaXaqaaiabd6gaUbqdcqGHris5aaaakiabcYcaSiaaxMaacaWLjaWaaeWaaeaacqaIXaqmaiaawIcacaGLPaaaaaa@5764@

where *m*_*pi *_denotes the number of signaling paths from input to output in which protein *i *is involved, *m*_*fi *_denotes the number of feedback loops including protein *i*, and *n *is the number of all proteins in the network. The more MPSs protein *i *is involved in, the more important protein *i *is for the signaling network. The value of *SigFlux *varies between 0 and 1. The extreme value of zero occurs when protein *i *is not a member of any MPS, and the extreme value of one is assigned to the most important protein in the network, i.e. the protein whose removal causes a disruption in the topological structure of the network.

To demonstrate the performance of *SigFlux*, we studied the signaling network in the hippocampal CA1 neuron of a mouse [13]. We collected the mutant phenotype and evolutionary rate of mouse genes to evaluate *SigFlux *[see Additional file 1].

### *SigFlux *is significantly correlated with a protein's essentiality

The functional significance of a gene is elementarily defined by its essentiality [12]. In simple terms, an essential gene is one whose removal renders the cell unviable. An effective experiment for evaluating the importance of genes in a cell or body is to mutate their phenotypes. Thus, to assess *SigFlux *as a measure of the importance of genes, we first sought to find the correlation between *SigFlux *and the essentiality of genes.

**Figure 1:**
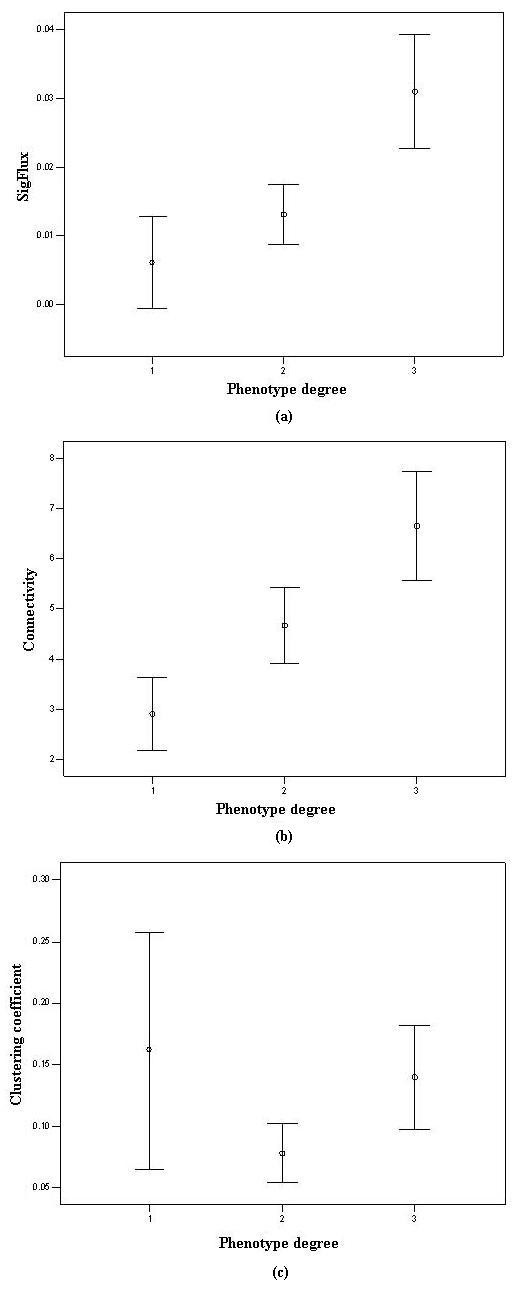
***SigFlux*, connectivity and the clustering coefficient of mouse proteins in different phenotype groups**. Mutant phenotypes of genes in mouse are grouped into the following 3 categories: group 1 corresponds to no obvious phenotype, group 2 is a viable phenotype, and group 3 is a lethal phenotype, as shown on the x-axis. The median and standard deviation of *SigFlux*, connectivity and the clustering coefficient in different groups are shown on the y-axis, respectively. (a) A positive correlation exists between *SigFlux *and essentiality. (b) A positive correlation exists between connectivity and essentiality. (c) No obvious correlation exists between the clustering coefficient and essentiality.

The relations between phenotype and *SigFlux*, as well as connectivity and clustering coefficient, are depicted in Figure 1. Proteins with higher *SigFlux *value tended to be more essential. That is, the essential proteins tended to participate in more signaling paths or feedback loops and played crucial roles in signal propagation than the other proteins. This result implies that *SigFlux *can, in some situations, describe to what extent that a given protein is involved in functions in a biological system. From this point, *SigFlux *shows the close relationship between network topology and function. This significant positive correlation between *SigFlux *and essentiality means *SigFlux *may be a promising index to predict the essentiality of corresponding genes. Also, highly connected proteins tended to be more essential. However, the clustering coefficient of genes has no obvious correlation with their essentiality in this signaling network.

We remark that although there was a high consistency between essentiality and *SigFlux*, some exceptions still exist. One case in point is that some proteins with an essential mutant phenotype cannot be involved in any MPS because these proteins have no global impact to the signaling network or the signaling network is not completely uncovered.

### *SigFlux *may act as the indictor of protein evolutionary rate

[18,19] reported that proteins with topological importance in networks and increased essentiality are likely to be conserved in evolution to preserve their functional stability. Therefore, proteins involved in more MPSs are supposed to evolve slower in principle since an MPS can be seen as the minimal function module. The relationship between *SigFlux *and evolutionary rate is investigated in this paper to validate this hypothesis.

As shown in Table 1, a significant negative correlation was found between *SigFlux *and evolutionary rate (Pearson r = -0.115, p = 0.008), which validates our hypothesis above. This implies proteins that participate in more MPSs are under greater evolutionary constraint to maintain their stability of function. This result demonstrates that *SigFlux *may be a proper network feature to assess proteins' importance in signaling networks. Moreover, we found a significant correlation between evolutionary rate and connectivity in signaling networks. This correlation was also reported by [19] in the context of protein interaction networks. Highly connected proteins are more conserved than their less-connected counterparts in signaling networks. However, we can not find obvious correlation between the clustering coefficient of a protein and its evolutionary rate. Hence, clustering coefficient appears not to be a proper network feature to evaluate the importance of proteins in signaling networks.

**Table 1:** Pearson correlation coefficients r (p-values) between three network features and the evolutionary rate of mouse genes.

	***SigFlux***	**Connectivity**	**Clustering coefficient**
**Evolutionary rate**	-0.115(0.008)	-0.136(0.002)	-0.019(0.662)

### Comparison of *SigFlux *with connectivity

Although we found that both *SigFlux *and connectivity were correlated with essentiality and evolutionary rate of mouse genes, they describe different topological features of signaling networks. The distribution of the connectivity across the nodes of the network has been used as a measure to characterize natural networks and was suggested to correlate with the importance of the protein. However, this is only valid if the immediate neighbors are the only ones determining the properties of a protein in the network. In contrast, *SigFlux *indicates how important the node is within the wider context of the entire network. Instead of its direct interactors, all correlative proteins between input layer and output layer are counted to determine a protein's importance in signaling networks. Thus, it seems reasonable to integrate *SigFlux *and connectivity to analyze the structural properties of signaling networks.

**Figure 2:**
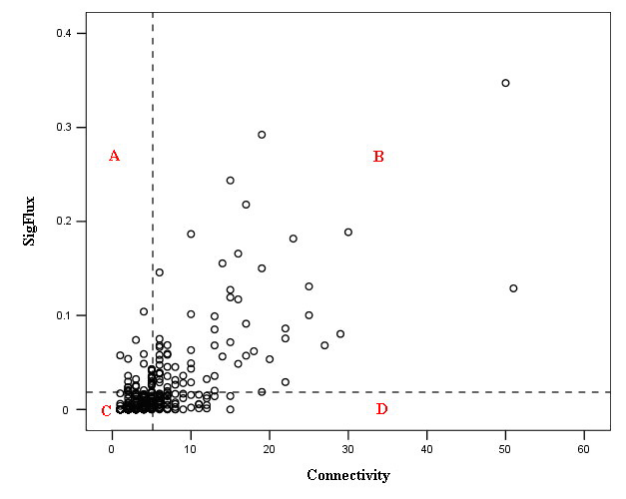
***SigFlux *is plotted as a function of connectivity**. High-*SigFlux *is assigned to proteins with above average *SigFlux *value, otherwise low-*SigFlux*. High-connectivity and low-connectivity proteins are defined in the same way. Region A includes all high-*SigFlux*, low-connectivity proteins, Region B high-*SigFlux*, high-connectivity proteins, Region C low-*SigFlux*, low-connectivity proteins, and Region D low-*SigFlux*, high-connectivity proteins.

**Figure 3:**
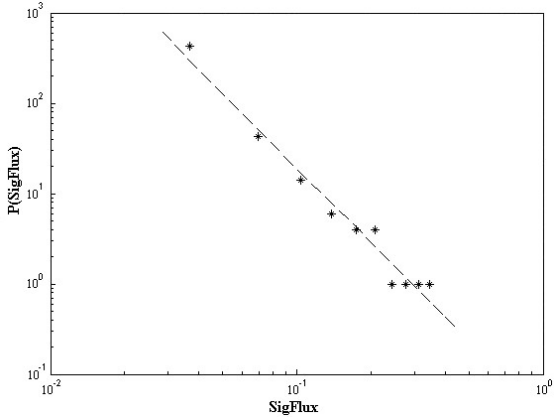
**The distribution of *SigFlux *follows a power law**. The value of *SigFlux *is divided into ten equal sections, and the number of proteins whose *SigFlux *locate in each section is counted. The distribution of *SigFlux *follows a power law, with *P*(*SigFlux*) ~ *SigFlux*^-*γ*^, *γ *= 2.72 ± 0.16 (p = 1.34 × 10^-7^).

**Figure 4:**
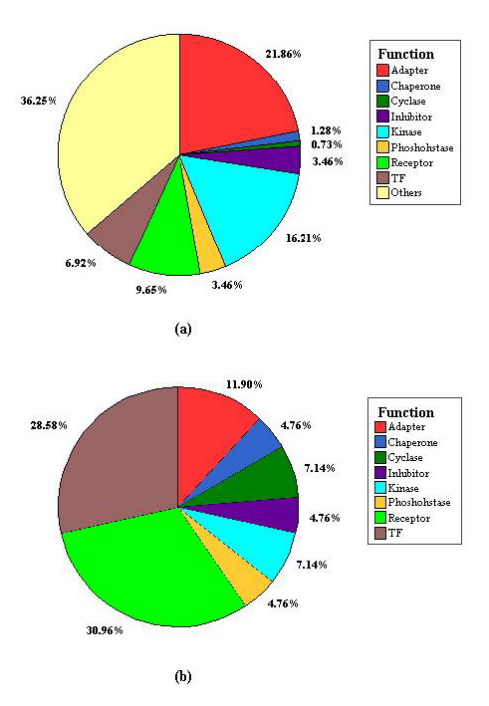
**Proteins are classified according to their function in the signaling network**. All proteins are classified according to their function in the signaling network as shown in (a), and HSLC proteins as shown in (b). Corresponding hypergeometric p-values of receptors and transcriptional factors are 4.486 × 10^-5 ^and 5.317 × 10^-6^, respectively. The result shows that HSLC proteins are abundant in receptors and transcriptional factors.

To compare *SigFlux *with connectivity, we depict the distribution of *SigFlux *as a function of connectivity in Figure 2. Here, proteins with different *SigFlux *and connectivity combinations are assigned to four different regions. Obviously the number of low-*SigFlux*, low-connectivity proteins is much larger than that of high-*SigFlux*, high-connectivity proteins. The probability *P*(*SigFlux*) that a protein has a certain *SigFlux *in the signaling network decays following *P*(*SigFlux*) ~ *SigFlux*^-*γ *^as shown in Figure 3, with *γ *= 2.72 ± 0.16 (p = 1.34 × 10^-7^). This indicates *SigFlux*'s distribution in the signaling network has a scale free property [20]. The few highly connected proteins have high *SigFlux *values because there are many nodes directly and exclusively connected to these proteins and the signaling paths go through these proteins. However, the existence of some nodes with high *SigFlux *but low connectivity (HSLC) implies that nodes with few interactors may have a global impact to the signaling network due to their high *SigFlux *value. We found that HSLC proteins are rich in receptors and transcriptional factors, as shown in Figure 4(b), with hypergeometric p-values of 4.486 × 10^-5 ^and 5.317 × 10^-6^. As a control, the function distribution of all proteins in signaling network is given in Figure 4(a). It is reasonable for them to possess a high *SigFlux *value owing to their special positions in signaling networks. Though parts of receptors do not interact with many proteins, they are the crucial intermediates to transfer signals from the extracellular ligands to specified effectors, such as FAS and TNFR1, etc. It is similar for some transcriptional factors that are less-connected but crucial to regulate the final target genes. From a topological point of view, HSLC proteins are positioned to connect regions of high clustering even though they have low connectivity. Low-*SigFlux*, high-connectivity proteins are not included in many signal paths but mostly in feedback loops. This indicates that these proteins may play a local role in regulating signal propagation.

## Discussion

The definition of MPSs is in principle similar to EMs in metabolic networks, but there are significant differences between them. In metabolic networks we are particularly interested in the reactions (edges) because they respond to enzymes that are subject to regulatory processes and can be knocked-out in experiments [2]. In contrast, in signaling networks we usually focus on the nodes since they are often knocked-out in experiments or medical treatment. Whereas an edge in signaling networks represents mostly a direct interaction between a pair of nodes with no mediator. An MPS in signaling networks is a set of proteins functioning together instead of enzymes in EM. From this point of view, MPS and EM methods have very different biological interpretations, although their mathematical definitions are similar to each other.

## Conclusion

In this paper we introduce a novel network feature *SigFlux *in signal transduction networks on the basis of the concept of minimal path sets. We found a significant correlation between *SigFlux *and the essentiality, as well as between *SigFlux *and the evolutionary rate of genes. These correlations held true for connectivity but not for the clustering coefficient. The comparison between *SigFlux *and connectivity implies that *SigFlux *and connectivity may both be useful features to measure the importance of proteins, although they describe the different topological properties of signaling networks. Further classification according to proteins' function demonstrated that HSLC proteins are abundant in receptors and transcriptional factors. This means that *SigFlux *may indicate some important proteins located at the connected regions of high clustering, even though they have low connectivity.

While in this paper we focused on signaling networks, the methods can be easily applied to any kind of interaction networks, such as gene regulatory networks. The final aim for analysis of network structural properties is to give some clues to better understand the function of the biological network. Insights into inherent properties of biological systems will provide us with a better understanding of complex diseases and a guiding principle for therapy design.

## Methods

### Signaling network in the hippocampal CA1 neuron of a mouse

We downloaded the signaling network in the hippocampal CA1 neuron of a mouse from the supplemental material of [13], including 608 biological molecules and 1427 interactions between them.

In this signaling network, three types of functional links are specified. Links may be activating, inhibitory or neutral. Some pathways activate a target gene while others inhibit it. For example, EGF (epidermal growth factor) [9,21] activates cell growth, survival or differentiation through some pathways and inhibits apoptosis through other pathways. Therefore, the networks can be separated and investigated respectively according to function as activators or inhibitors to a target node. But in the computation of the network feature we propose above, activating and inhibitory paths are not distinguished and both seen as MPSs.

### The method to generate all the MPSs

**Figure 5:**
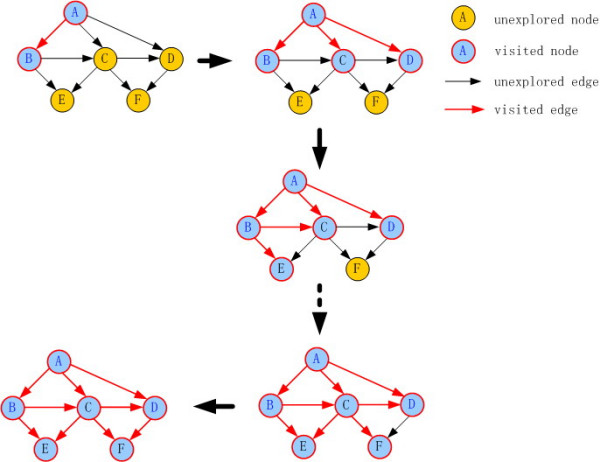
**A simple example is shown to illustrate how breadth-first search method works**. Given a network G, first label all the nodes of G and get its parent index. Then generate paths starting from a root node. The nodes with shorter distance to the root node are explored preferentially. Paths starting from root to target are returned when all the nodes are visited. In the network below, take A as the root node and E, F as target nodes. The resulting path sets are {A,B,E}, {A,C,E}, {A,B,C,E}, {A,C,F}, {A,D,F}, {A,C,D,F}, {A,B,C,F} and {A, B,C,D,F}.

According to the function and subcellular location of nodes, 33 nodes functioning as an extracellular ligand and matrix are defined as an input layer, and 20 nodes including DNA/RNA and a part of transcriptional factors as an output layer. First, all the paths between the input layer and output layer, including activating and inhibitory pathways, were generated using the classical breadth-first search method [15]. In graph theory, breadth-first search is a graph search algorithm that aims to expand and examine all nodes of a graph systematically in search of a solution. In Figure 5, a simple example is given to illustrate how the breadth-first search method works. There are a large amount of alternate paths between input and output, usually thousands of paths, which are derived from the diverse nature of signaling pathways [22]. To improve the efficiency of computation, we constrain the maximal size of pathways to 20 nodes and generate the shortest paths preferentially. It is reasonable to restrict the size of all pathways within 20 nodes since the typical length of real signaling pathways is between 7 and 14 [13]. It is found that *SigFlux *changes slowly once the number of generated paths exceeds a certain value. Thus it is efficient and effective to only generate a large part of paths, i.e. enough to reach a relative stable value of *SigFlux*, instead of enumerating all paths in large signaling networks. Then, we identified feedback loops using the MFinder program. MFinder is a software tool for network motifs detection, in which network motifs are defined as patterns of interconnections occurring more frequently than in randomized networks. The MFinder program searches for motifs in directed networks, but it does not distinguish positive and negative links. As a result, the pathways number from the input layer to the output layer is 297,397 and the number of feedback loops is 4,078.

### Phenotype and evolutionary rate of mouse genes

Since there are rich data about mutant phenotype of mouse genes and evolutionary information between mouse and orthologous genes, we can easily download them from a public database, such as MGD [23] and the Ensembl Gene database [24].

After removing lipids, messengers and Ions etc. that have no corresponding genes from the set of nodes in the mouse signal transduction network, there are 549 proteins reserved for investigation. Of these, the mutant phenotype of 383 genes are found in MGD, including 34 genes with no obvious phenotype, 191 with viable phenotype and 158 with lethal phenotype. "Lethal" refers to perinatal lethal, postnatal lethal or embryonic lethal; "viable" phenotypes lead to abnormal response or illness but no death; "no obvious phenotype" are mice for which disruptions of this gene display a normal phenotype. There are still 166 proteins with genetic phenotypic information unavailable due to the lack of gene mutation experiments or data not stored in MGD. According to the mutant phenotype of mouse genes, the essentiality of proteins in signaling networks is grouped into 3 categories: no obvious, viable and lethal phenotypes.

To build a source of orthologous data, we browsed gene clusters compiled in the Ensembl Gene database, which provides evolutionary information of orthologous gene pairs including mouse and other eukaryotes. Using this, we downloaded 525 proteins' dN/dS [25] between Mus musculus and their orthologous gene pairs in Homo sapiens, Bos Taurus, Pan troglodytes, Macaca mulatta and Canis familiaris. dN/dS is defined as the nonsynonymous rate divided by the number of synonymous differences per synonymous site. Usually dS is an estimate of the neutral rate of molecular evolution. Then, investigating dN/dS may provide information about the degree of selection operating on a species. Therefore, average dN/dS between mouse genes and their orthologous ones in other five species can represent the evolutionary rate of mouse genes to some extent.

## List of abbreviations

EM(s): elementary flux mode(s)

MPS(s): minimal path set(s)

HSLC: high *SigFlux*, low connectivity

## Authors' contributions

W.L. conceived of the study, wrote the program code and drafted the manuscript. D.L. participated in the design of the study and contributed to the methods' development. JY. Z. wrote part of the program and revised the manuscript critically. YP.Z. gave excellent advice and assistance to the process of project. FC.H. guided the study and coordinated the project. All authors read and approved the final manuscript.

## Supplementary Material

Additional File 1 Genes, Mouse Swiss-Prot ID, MGI, mutant phenotype etc. This file includes mutant phenotype and evolutionary rates of mouse genes and also provides *SigFlux*, connectivity, and the clustering coefficients of all proteins in the signaling network. Click here for file
